# Multiple Defects Impair the HLA Class II Antigen Presentation Capacity of Burkitt Lymphoma

**DOI:** 10.4172/2155-9899.1000e119

**Published:** 2016-08-05

**Authors:** Jason M. God, Azizul Haque

**Affiliations:** Department of Microbiology and Immunology, and Hollings Cancer Center, Medical University of South Carolina, Charleston, USA

**Editorial**

Burkitt lymphoma (BL) is a B-cell malignancy which occurs with varying clinical manifestations and frequencies. The most well-known presentation of BL is found in areas which are holoendemic or hyperendemic for malaria. This endemic BL occurs primarily in children and presents as tumors of the jaw [1,2]. Though a strong association exists between endemic BL and malaria, the nature of the relationship remains unclear. A sporadic form of BL also occurs elsewhere in the world and typically causes tumors in the gut and upper respiratory tract [3]. There are various other contributing factors which may lead to development of BL, including Epstein-Barr Virus (EBV) and Human Immunodeficiency Virus (HIV) [4,5]. EBV has long been associated with development of BL and is found in nearly all cases of endemic BL but, in spite of intense research, the relationship between EBV and BL remains unclear. It is generally believed that infection with EBV somehow drives transformation which then leads to development of BL [6]. While the exact cause of BL has remained elusive (and is likely multi-factorial) the hallmark of all BL is the constitutive activation of the *MYC* gene resulting in its overexpression and cell transformation [7,8]. Most commonly, this occurs through translocation of the *MYC* gene to an immunoglobulin locus, which in B cells would result in constitutive expression [8]. Though translocation of *MYC* was long considered a diagnostic criterion for BL, recent evidence suggests other mechanisms may be responsible for *MYC* deregulation in a small percentage of BL cases [9].

BL is a rapidly growing malignancy with a doubling time of 24 hours and consequently requires aggressive therapies for treatment [10]. While cure rates can approach 80%, the aggressive treatments are not well tolerated by the elderly or the immunocompromised. Thus, for these patient populations in particular, investigation into alternative therapies which are less toxic is of paramount importance. Of particular interest are approaches which harness the immune system’s potent anti-tumor responses to specifically target BL malignancies. BL possesses a well-known deficiency in its ability to stimulate immune responses through human leukocyte antigen (HLA) class I as a result of poor immunogenicity associated with EBV nuclear antigen 1 (EBNA1) [11]. As a result of this defect, CD8^+^ T-cell activation is weak and lasting cellular immunity is not generated. The HLA class II response remains an area of interest, however, as antigen presentation through class II molecules, resulting in activation of CD4^+^ T-cells, has been shown to enhance and sustain cytotoxic T lymphocyte (CTL) immunity [12]. While the HLA class I defect is well-known, the underlying defect in class II presentation remains uncertain. If it were possible to shed light on the cause of this defect, it may be possible to generate immunotherapies which would allow for effective presentation of antigen through HLA class II, resulting in tumor destruction. With this goal in mind, our laboratory has focused on unraveling the complexities associated with the lack of a HLA class II response to BL malignancies [13,14].

To explore this, we employed several strategies in the hopes of isolating inhibitory molecules which may be implicated in the HLA class II antigen presentation defect. We have employed several cell lines for the purpose of assaying CD4^+^ T-cell activation: BL lines Nalm-6, Ramos and Ous, and B-LCL lines Frev and 6.16.DR4.DM. Cell lines were retrovirally transduced to generate: Ramos.DR4, Nalm-6.DR4, and 6.16.DR4.DM; Ous and Frev constitutively express HLA-DR4 molecules. Surface expression of HLA-DR4 was confirmed in all cell lines by flow cytometry [11]. Thus, all cell lines used expressed a common allele of HLA class II. Early studies established that BL lines (Nalm-6.DR4 and Ramos.DR4) were defective in their ability to stimulate CD4^+^ T-cells through the HLA-DR4 class II molecule while 6.16.DR4.DM generated a strong CD4^+^ T-cell response [13,14]. Peptide binding was confirmed in all cell lines under neutral (pH 7.4) and acidic (pH 5.5) conditions. It was, however, noted that Nalm-6.DR4, Ramos.DR4 and 6.16.DR4.DM all stimulated high levels of IL-2 production when antigen presentation assays were carried out under pH 5.5 conditions. When carried out under pH 7.4 conditions, results were similar to those previously observed (BL= low IL-2, B-LCL=high IL-2). This led us to evaluate the eluate from the different incubation conditions (pH 7.4 *vs* pH 5.5). When antigen presentation assays with 6.16.DR4.DM were performed in the eluate obtained from incubating BL cells in pH 5.5 buffer, it was discovered that the >30 kDa fraction of the pH 5.5 eluate drastically impaired the antigen presentation ability of 6.16.DR4.DM. Further, this impairment was ablated by treating the eluate with proteinase K. Taken together, this suggests the presence of Burkitt Lymphoma-Associated Inhibitory Molecule (BLAIM) which is proteinaceous in nature.

In an effort to shed light on the identity of BLAIM, we next compared protein expression patterns between Frev, Nalm-6.DR4 and 6.16.DR4.DM cells. pH 5.5 eluates obtained from each cell line were separated on a non-reducing gel and the gel was then stained with Coomassie blue. A comparison of the banding pattern revealed a ~50 kDa protein which was highly expressed in Frev and 6.16.DR4.Dm but absent or only slightly expressed in Nalm-6.DR4. Extraction and mass spectrophotometric analysis of this protein band revealed a 47 kDa enolase-like protein [14]. Further studies revealed that this protein gel extract was capable of stimulating much higher IL-2 responses in B-LCL but not in BL. Though our efforts in comparing protein expression patterns between BL and B-LCL were done with the hopes of identifying BLAIM, we instead uncovered another possible BL defect with the decreased expression of the immunostimulatory 47 kDa enolase-like protein. Thus, it seems increasingly likely that BL’s inability to generate an HLA class II response is multi-faceted and likely involves the increased expression of inhibitory molecules (e.g. BLAIM) concomitant with the decreased expression of immunostimulatory molecules (e.g. 47 kDa enolase-like protein).

In support of our idea that the defect in HLA class II antigen presentation is multifactorial, we have previously shown that cells in which *c-MYC* is overexpressed display a reduced capacity to present antigen via HLA class II molecules [15]. To investigate this, we evaluated the surface and intracellular levels of HLA-DR4 and class II pathway processing components through Western blotting and flow cytometry in c-MYC^high^ and c-MYC^low^ cells. Our results demonstrated that while surface expression of HLA class II is not decreased in c-MYC^high^ cells when compared to c-MYC^low^ cells, the intracellular expression of two critical components of the class II pathway were sharply decreased. We observed a significant decrease in the expression of HLA-DM and GILT (a lysosomal reductase) in c-MYC^high^ cells when compared to c-MYC^low^ cells. HLA-DM and GILT are both essential for the efficient and proper processing of antigens for presentation via the HLA class II pathway. It logically follows that decreasing the expression levels of either one of these molecules would have deleterious effect on the ability of cells to present antigens via class II molecules [15].

Thus, we have demonstrated that BL cells appear to have multiple points of defect in their ability to effectively present antigen through the HLA class II pathway. The process of antigen presentation is hampered both at the level of antigen processing, resulting from decreased levels of class II pathway antigen processing components, and at the level of antigen presentation, through BLAIM and the 47 kDa enolase-like protein which appear to interfere directly with presentation. Ongoing and future studies focus on a more detailed characterization of BLAIM and the 47 kDa enolase-like protein. Additionally, we will continue to explore the link between *c-MYC* overexpression and decreased expression of class II pathway processing components. A greater understanding of either the presentation or processing defects could lead to the development of novel immunotherapies which could be used in support of traditional chemotherapy, or could eliminate the need for chemotherapy altogether. The development of alternative and less toxic therapies is a necessary endeavor for those patient populations who do not tolerate well the aggressive treatments traditionally used for BL. Further, given the similarities among many cancers, we are hopeful that our work with BL will provide insights which may lead to a greater understanding of the immunological aspects of cancer development.

## References

[1] Magrath I (2012). Epidemiology: clues to the pathogenesis of Burkitt Lymphoma. Br J Haematol.

[2] Yustein J, Dang C (2007). Biology and treatment of Burkitt Lymphoma. Curr Opin Hematol.

[3] Pagano L, Caira M, Valentini C, Fianchi L (2009). Clinical aspects and therapy of sporadic burkitt lymphoma. Mediterr J Hematol Infect Dis.

[4] Grömminger S, Mautner J, Bornkamm G (2012). Burkitt lymphoma: the role of Epstein-Barr virus revisited. Br J Haematol.

[5] Rowe M, Fitzsimmons L, Bell A (2014). Epstein-Barr virus and Burkitt lymphoma. Chin J Cancer.

[6] Shatzer A, Espey M, Chavez M, Tu H, Levine M (2013). Ascorbic acid kills Epstein –Barr virus positive Burkitt lymphoma cells and Epstein-Barr virus transformed B-cells in vitro, but not in vivo. Leuk Lymphoma.

[7] Said J, Lones M, Yea S (2014). Burkitt lymphoma and MYC: what else is new?. Adv Anat Pathol.

[8] Gerbitz A, Mautner J, Geltinger C, Hörtnagel K, Christoph B (1999). Deregulation of the proto-oncogene c-myc through t(8;22) translocation in Burkitt’s lymphoma. Oncogene.

[9] De Falco G, Ambrosio M, Fuligni F, Onnis A, Bellan C (2015). Burkitt lymphoma beyond MYC translocation: N-MYC and DNA methyltransferases dysregulation. BMC Cancer.

[10] Ott G, Rosenwald A, Campo E (2013). Understanding MYC-driven aggressive B-cell lymphomas: pathogenesis and classification. Blood.

[11] Masucci M, Torsteindottir S, Colombani J, Brautbar C, Klein E (1987). Down-regulation of class I HLA antigens and of the Epstein-Barr virus-encoded latent membrane protein in Burkitt lymphoma lines. Proc Natl Acad Sci U S A.

[12] Khanolkar A, Badovinac V, Harty J (2007). CD8 T cell memory development: CD4 T cell help is appreciated. Immunol Res.

[13] Amria S, Cameron C, Stuart R, Haque A (2008). Defects in HLA class II antigen presentation in B-cell lymphomas. Leuk Lymphoma.

[14] God J, Zhao D, Cameron C, Amria S, Bethard J (2014). Disruption of HLA class II antigen presentation in Burkitt lymphoma: implication of a 47,000 MW acid labile protein in CD4+ T-cell recognition. Immunology.

[15] God J, Cameron C, Figueroa J, Amria S, Hossain A (2015). Elevation of c-MYC disrupts the HLA class II-mediated immune recognition of human B cell tumors. J Immunol.

